# Supplementary material for: Fate of telomere entanglements is dictated by the timing of anaphase midregion nuclear envelope breakdown
Source: Nat Commun. 2024 Jun 3;15:4707. doi: 10.1038/s41467-024-48382-2 (PMC11148042; doi:10.1038/s41467-024-48382-2)
Supplement: Supplementary file 1 — Supplementary Information [file 41467_2024_48382_MOESM1_ESM.pdf]

Figure S1

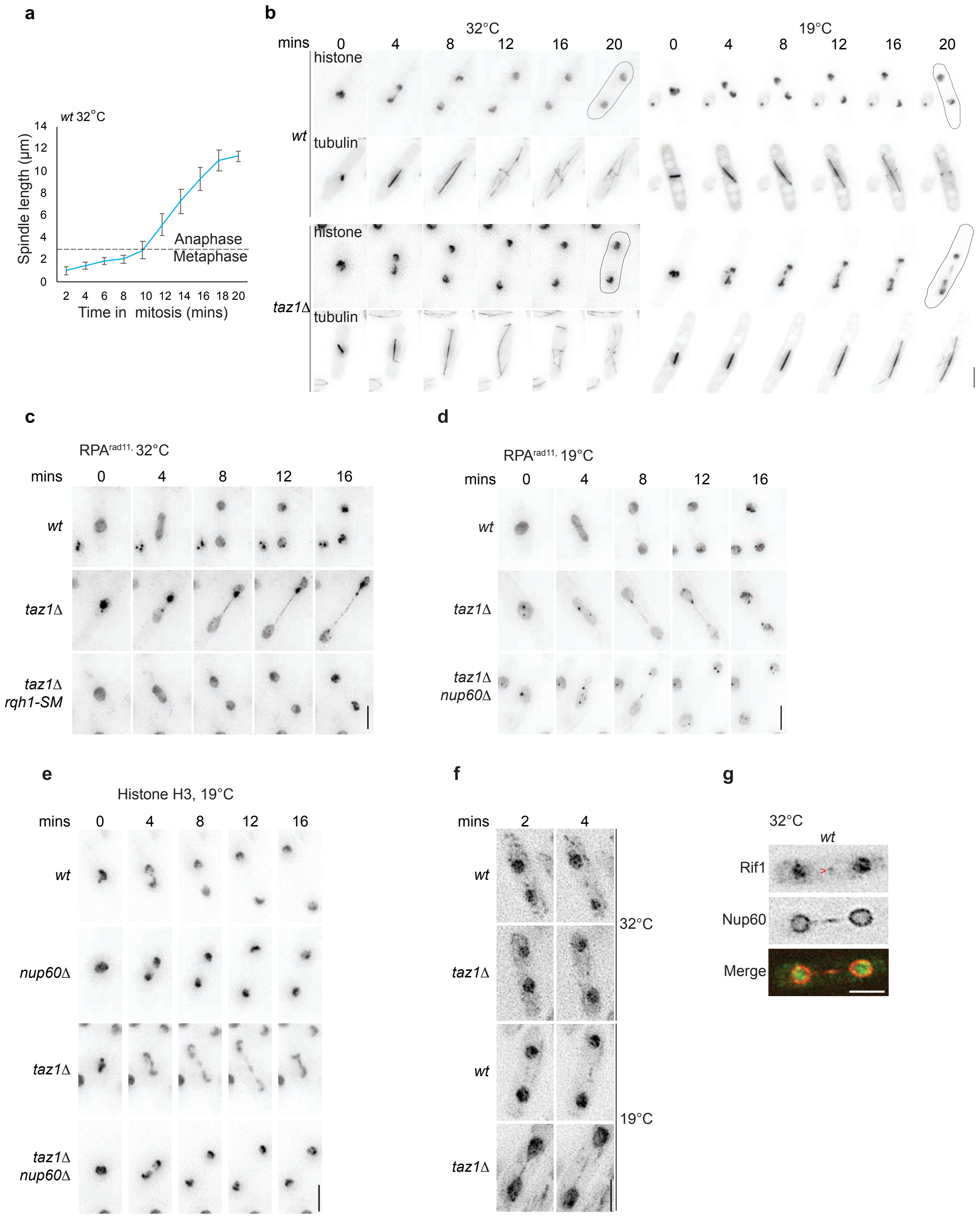

a
b
wt 32°C
Spindle length (μm)
Time in mitosis (mins)
Anaphase
Metaphase
32°C
19°C
mins
histone
tubulin
wt
taz1Δ
c
RPA[rad11], 32°C
d
RPA[rad11], 19°C
rqh1-SM
nup60Δ
e
Histone H3, 19°C
f
g
Rif1
Nup60
Merge

Figure S1: **Nuclear pore complex components hinder telomere detanglement. *a-b***, Graph representing mitotic spindle dynamics in *wt* cells expressing an ectopic copy of GFP-α-tubulin at the *aur1+* locus. Spindle length (μm) was measured from the onset of spindle formation until disassembly, and plotted against the corresponding time. The base of the exponential spindle elongation (3μm) is used as the time of exit from metaphase throughout this study. Mean spindle length ± standard deviation is represented against time in mitosis. **b**, Frames from films shown in Figure 1c including the corresponding spindle images for each frame. *wt* (at 32ºC and 19ºC) and *taz1Δ* (at 32ºC) show similar spindle dynamics; on the contrary, *taz1Δ* cells maintained at 19ºC show longer and hyperstable spindles, which are delayed in disassembly. **c**, Frames from representative films of RPA^{Rad11} in mitotic cells of the indicated genotypes; these correspond to the graphs in Figure 1d. *rqh1-SM* suppresses the formation of aberrant RPA bridges in a *taz1Δ* setting. The cells were maintained in log-phase at 32ºC. Time 0 represents metaphase, as described in Figures S1a. **d and e**, Frames from films of mitotically dividing cells maintained in log phase at 19ºC for 3 days before imaging RPA or histone H3, respectively. **f**, Frames from films of mitotically dividing cells expressing endogenously C-terminally tagged Rif1-GFP. Time indications start at an arbitrary point in early mitosis. **g**, A mitotically dividing cell in anaphase with endogenously tagged Nup60-mCherry and Rif1-GFP.

Figure S2

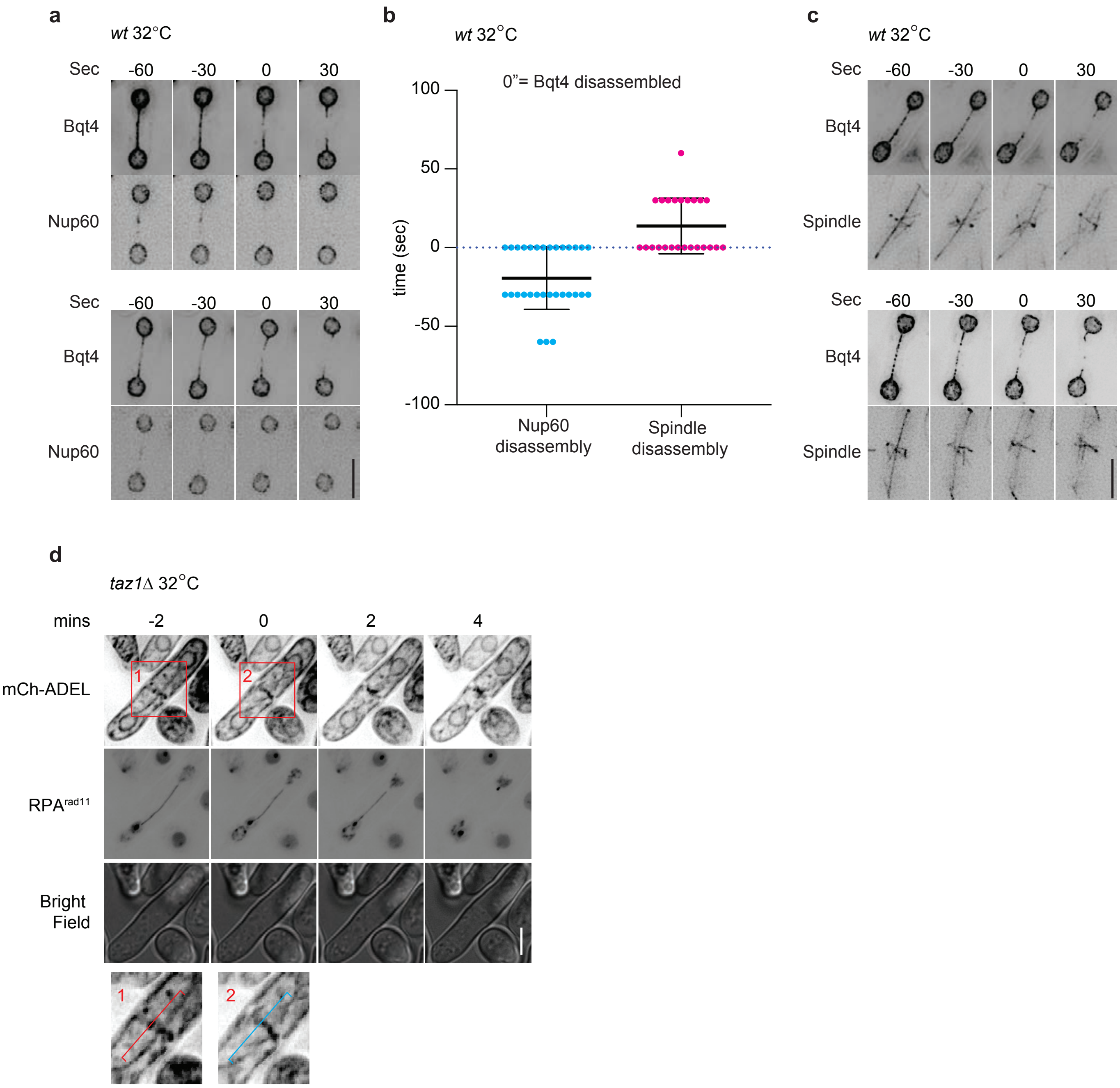

a
wt 32°C
Sec
-60
-30
0
30
Bqt4
Nup60
b
wt 32°C
0"= Bqt4 disassembled
100
50
0
-50
-100
time (sec)
Nup60 disassembly
Spindle disassembly
c
Spindle
d
taz1Δ 32°C
mins
-2
2
4
mCh-ADEL
RPArad11
Bright Field
1
2

Figure S2: Entanglements are in the cytoplasm at the time of resolution. **a**, Frames from films of mitotically dividing *wt* cells with endogenously tagged Nup60-mCherry and Bqt4 visualized via an additional *bqt4+* copy at the *lys1+* locus under the cognate *bqt4+* promoter, aquired every 30 seconds. **b**, Graph shows the timing of NPC disassembly and the onset of spindle disassembly relative to Bqt4 disassembly. Mean time in seconds ± standard deviation is represented. **c**, Frames from films of mitotically dividing *wt* cells expressing GFP-Bqt4 as in Figure S2a and mCherry-atb2 (encoding α-tubulin, as in Figure 1b) aquired every 30 seconds. **d**, Frames from films of mitotically dividing *taz1Δ* cells grown at 32ºC expressing mCherry-ADEL (NE/ER marker) from the *lys1+* locus and endogenously tagged Rad11-GFP.The lower panel shows the insets indicated by the red boxes. The brackets indicate the NE (left, in red) and local NE breakdown (right, in blue), around the RPA^{Rad11} bridge. 0 minutes in this figure (a, c and d) corresponds to when Bqt4 (in figure a-c) or mCherry-ADEL (in figure d) signal becomes discontinuous.

Figure S3

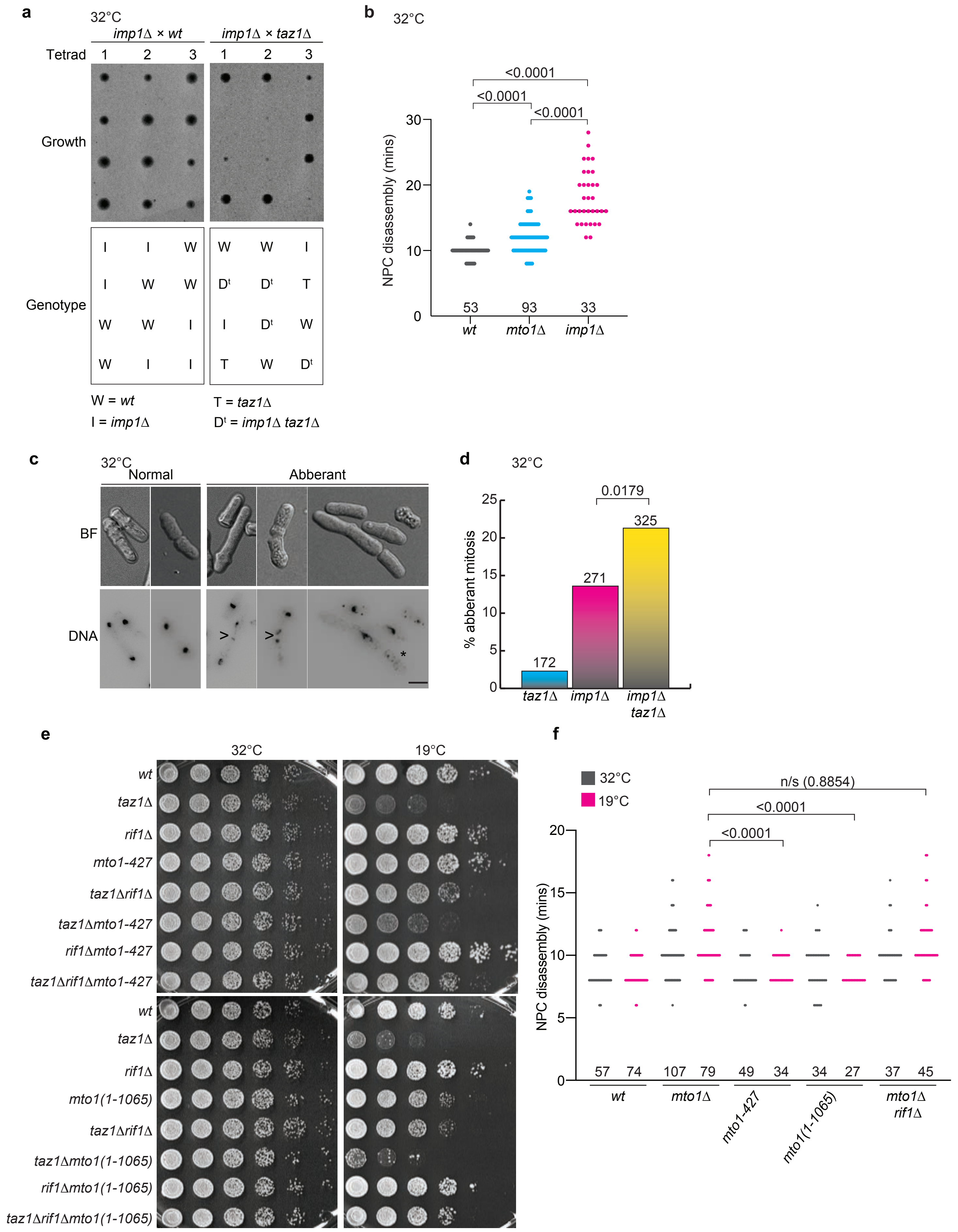

a
32°C
imp1Δ × wt
imp1Δ × taz1Δ
Tetrad
1 2 3
1 2 3
Growth
Genotype
I I W
I W W
W W I
W I I
W W I
Dt Dt T
I Dt W
T W Dt
W = wt
I = imp1Δ
T = taz1Δ
Dt = imp1Δ taz1Δ
b
32°C
NPC disassembly (mins)
<0.0001
<0.0001
<0.0001
53 93 33
wt mto1Δ imp1Δ
c
32°C
Normal
Abberant
BF
DNA
d
32°C
% abberant mitosis
0.0179
172 271 325
taz1Δ imp1Δ imp1Δ taz1Δ
e
32°C
19°C
wt
taz1Δ
rif1Δ
mto1-427
taz1Δrif1Δ
taz1Δmto1-427
rif1Δmto1-427
taz1Δrif1Δmto1-427
wt
taz1Δ
rif1Δ
mto1(1-1065)
taz1Δrif1Δ
taz1Δmto1(1-1065)
rif1Δmto1(1-1065)
taz1Δrif1Δmto1(1-1065)
f
32°C
19°C
n/s (0.8854)
<0.0001
<0.0001
NPC disassembly (mins)
57 74 107 79 49 34 34 27 37 45
wt mto1Δ mto1-427 mto1(1-1065) mto1Δ rif1Δ

Figure S3: Delayed cytosolic exposure hinders entanglement resolution: **a**, tetrad analysis of the indicated genotypes. An ascus from the heterogyzous cross was dissected and incubated at 32°C for 3 days. The upper panel shows growth and the lower panel shows the corresponding spore genotypes. *taz1Δimp1Δ* spores yield compromised viability. **b**, The timing of NPC disassembly is plotted relative to anaphase onset as in Figure 2e. Exact P values from two tailed Mann-Whitney test are indicated above brackets. n values are represented above each genotype. **c**, Bright field (BF) and DAPI-stained images of representative *taz1Δimp1Δ* cells derived from a, grown in liquid media, fixed and stained to monitor chromosome segregation. Arrowheads indicate examples of chromosome missegregation; asterisk indicates an enucleated cell. **d**, Quantitation of phenotypes shown in c. Exact P values derived by two tailed Fisher's exact T test are represented. n values represented over each bar. **e**, 5-fold serial dilutions were incubated at 32ºC (2 days) or 19ºC (7 days). **f**, The timing of NPC disassembly is plotted relative to anaphase onset for the indicated genotypes maintained in log phase at 32ºC (1 day) or 19ºC (3 days). For comparison, *wt* and *mto1*Δ data are replotted from Figure 4c. Exact P values derived from Mann-Whitney test are indicated above the brackets. n values are represented above each genotype.

Figure S4

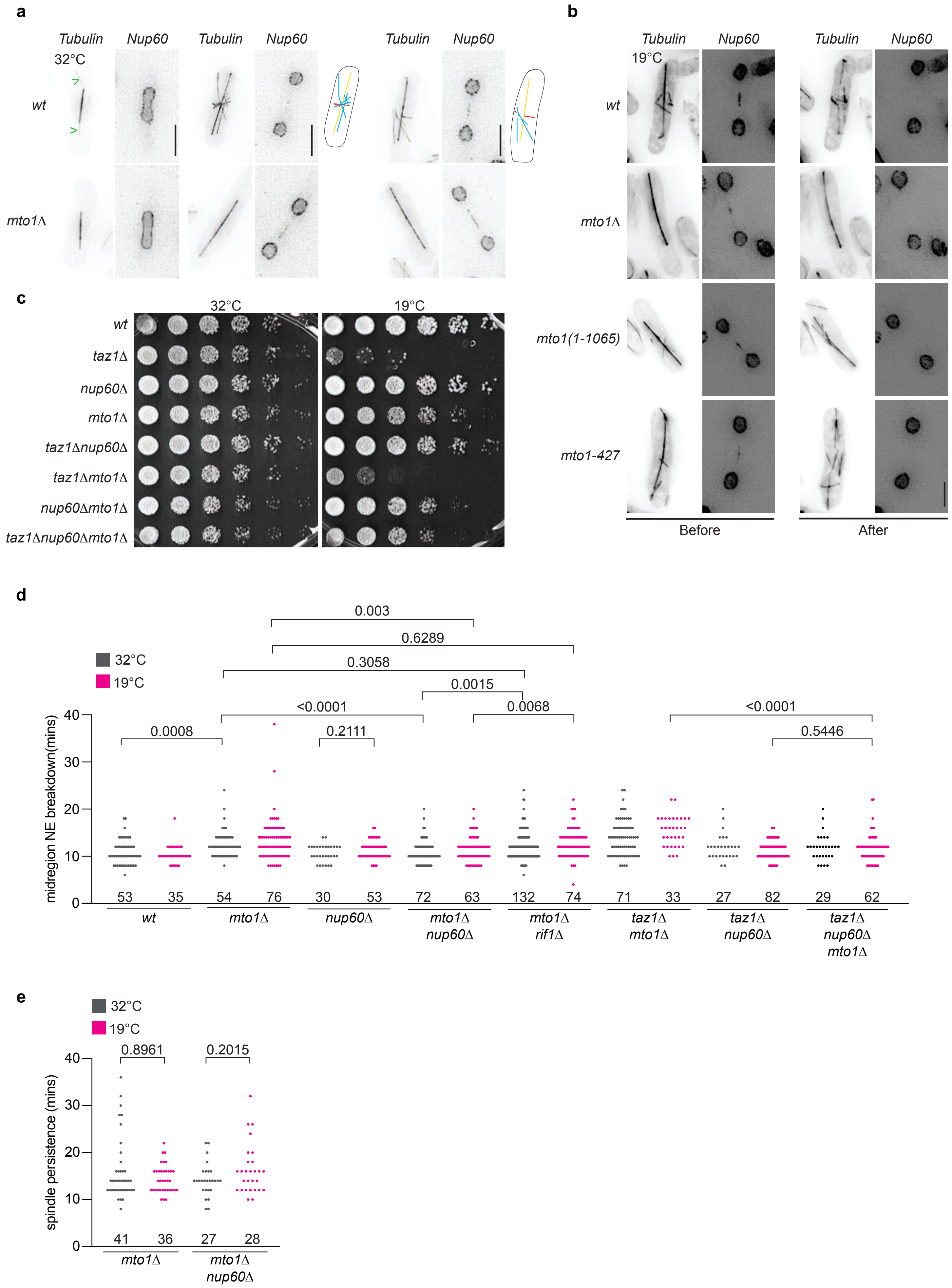
a
Tubulin
Nup60
32°C
wt
mto1Δ
b
19°C
mto1(1-1065)
mto1-427
Before
After
c
32°C
19°C
taz1Δ
nup60Δ
taz1Δnup60Δ
taz1Δmto1Δ
nup60Δmto1Δ
taz1Δnup60Δmto1Δ
d
midregion NE breakdown(mins)
0.003
0.6289
0.3058
0.0015
<0.0001
0.0068
0.0008
0.2111
0.5446
53
35
54
76
30
53
72
63
132
74
71
33
27
82
29
62
mto1Δ nup60Δ
mto1Δ rif1Δ
taz1Δ mto1Δ
taz1Δ nup60Δ
taz1Δ nup60Δ mto1Δ
e
spindle persistence (mins)
0.8961
0.2015
41
36
27
28

Figure S4: **Delayed midregion NE breakdown, not spindle persistence, of *mto1*Δ cells inhibits telomere entanglement resolution**. **a**, Frames from films of mitotic cells with Nup60-mCherry and GFP-tubulin grown at 32ºC. Arrowheads represent astral cytosolic microtubules nucleated from the SPB during mitosis. Cartoons representing the microtubules are shown to the right of each cell. Spindles are shown in yellow, equatorial microtubules in red, and post anaphase arrays in blue. In *wt* cells, equatorial and post anaphase arrays cross across the anaphase midregion. All the foregoing cytosolic microtubules types are absent in the absence of Mto1. **b**, Frames from films of mitotically dividing cells expressing Nup60-mCherry and GFP-tubulin before and after midregion Nup60-disassembly. The cells were maintained in the log-phase at 19ºC for 3 days before imaging. NE prodding MTs are present in *wt* and *mto1+* separation-of-function mutants, but are absent in *mto1Δ* cells. **c**, 5-fold serial dilutions were incubated at 32ºC (2 days) or 19ºC (7 days). **d**, The timing of anaphase midregion NE breakdown is plotted as minutes post-anaphase onset as indicated by the loss of NLS-GFP-βGAL signal from the midregion in cells maintained in log phase at 32ºC or 19ºC (as in Figure 2F). **e**, Timing of spindle disassembly relative to anaphase onset. Cells were maintained in log-phase at 32ºC or 19ºC. Exact P values derived from Mann-Whitney test are indicated above the brackets in d and e. n values are represented above each genotype.

Figure S5

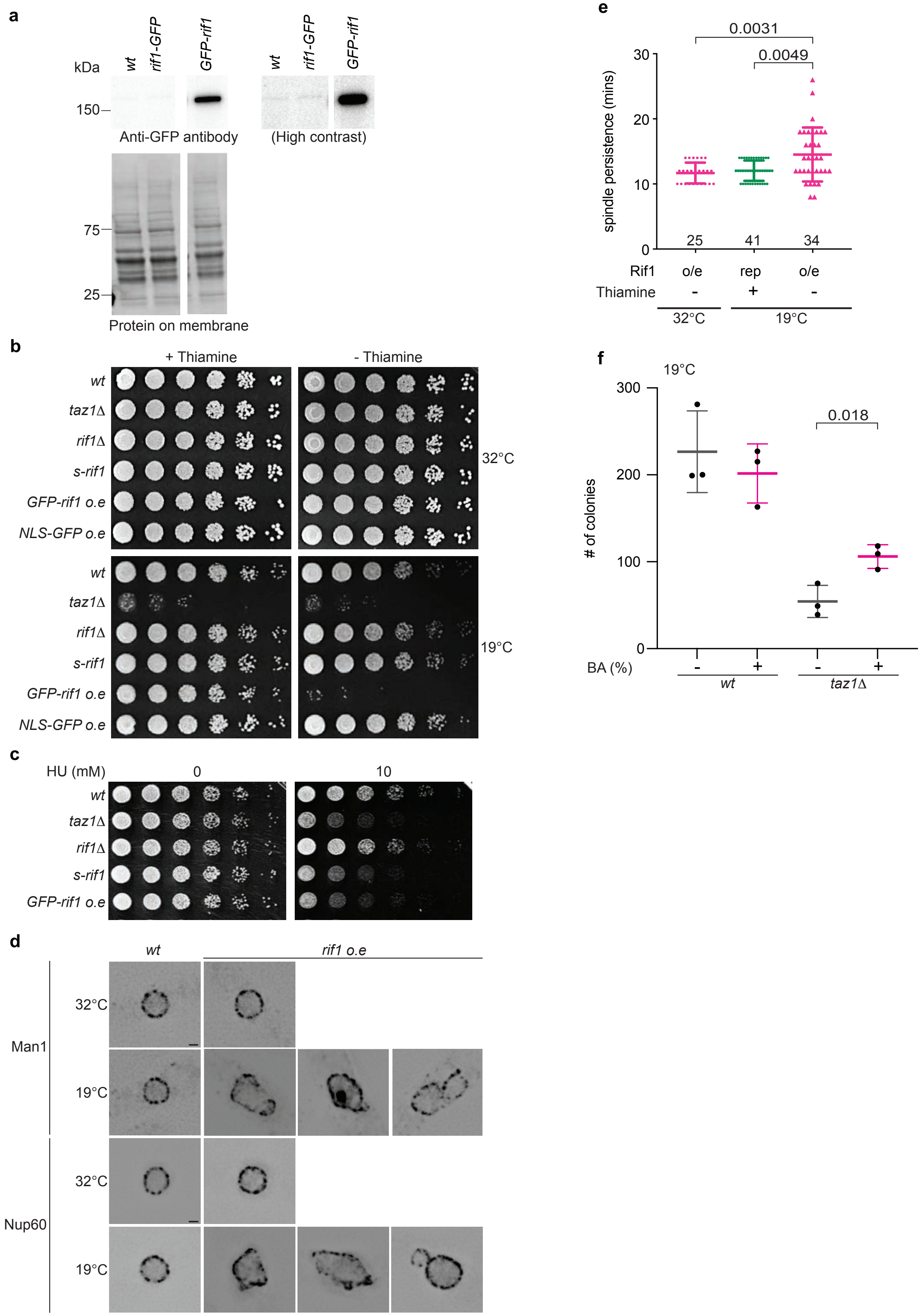

a
kDa
wt
rif1-GFP
GFP-rif1
150
Anti-GFP antibody
(High contrast)
75
25
Protein on membrane
b
+ Thiamine
- Thiamine
wt
taz1Δ
rif1Δ
s-rif1
GFP-rif1 o.e
NLS-GFP o.e
32°C
19°C
c
HU (mM)
0
10
d
wt
rif1 o.e
Man1
Nup60
32°C
19°C
e
0.0031
0.0049
spindle persistence (mins)
30
20
10
0
25
41
34
Rif1
o/e
rep
Thiamine
32°C
19°C
f
19°C
300
200
100
0
0.018
# of colonies
BA (%)
wt
taz1Δ

Figure S5: **Rif1 overexpression causes cold-specific, lethal NE deformities. a**, Western blot analysis of Rif1 using anti-GFP antibody. Cells were grown in PMG (without thiamine, to induce expression of *nmt41-gfp-rif1+*) media for 20 hours at 32ºC, total protein was TCA extracted and resolved on gradient SDS-PAGE. Endogenously tagged Rif1-GFP could not be detected. In contrast, overexpression of GFP-Rif1 from the *nmt41* (*no message in thiamine*) promoter yields a clear band at the expected molecular weight. Note that endogenously tagged Rif1-GFP is visible by microscopy (Figure S1f and g) and behaves as *rif1+* in *taz1Δ* cells, as reported previously[8]. **b**, 5-fold serial dilutions of cells grown to log phase in PMG minimal media with/without thiamine (which represses *nmt41-rif1+* expression) at 32ºC were incubated at 32ºC (2 days) or 19ºC (10 days). **c**, 5-fold serial dilutions of cells grown in PMG without thiamine to log phase at 32ºC were stamped on plates lacking thiamine, with or without 10mM hydroxyurea (HU); the plates were incubated at 32ºC or 19°C for 4 or 2 days, respectively. Cells overexpressing Rif1 are hypersensitive to HU. **d**, Images of cells with or without overexpressed GFP-Rif1; the cells also expressed either Man1-tomato or Nup60-mCherry from their respective endogenous loci. Each image is a single Z slice with the maximum signal intensity. Scale bars represent 1μm. Rif1 overexpression causes cold-specific nuclear envelope irregularities. **e**, Timing of spindle disassembly relative to anaphase onset. Cells were maintained in log phase in PMG minimal media with (repressed) or without (de-repressed) thiamine. Rif1 overexpressing cells are delayed in midregion NE breakdown and spindle disassembly only when grown in the cold. Exact P values from two-tailed Mann-Whitney test are indicated above brackets. **f**, Quantitation of colonies formed on plates at 32ºC (without BA) from cultures grown with 0% or 0.075% BA at 19ºC for 3 days. Each data point is an average of colonies formed from 3 technical replicates in which 300 cells were plated for each replicate. An average of three independent experiments is represented. Exact P-value derived from two-tailed parametric t-test is indicated above the bracket.

Table S1: List of strains used in the current study:

JCF #	Genotype	Origin
108	h- ade6-M210 his3-D1 leu1-32 ura4-D18	Lab stock
109	h+ ade6-M216 his3-D1 leu1-32 ura4-D18	Lab stock
15404	h+ taz1::hyg ade6-M216 his3-D1 leu1-32 ura4-D18	
15375	h- rif1::nat ade6-M210 his3-D1 leu1-32 ura4-D18	
13546	h- rif1:nat s-rif1 ade6-M210 his3-D1 leu1-32 ura4-D18	Lab stock
15408	h- taz1::hyg rif1::nat ade6-M his3-D1 leu1-32 ura4-D18	
15412	h+ taz1::hyg rif1:nat s-rif1 ade6-M his3-D1 leu1-32 ura4-D18	
15416	h+ mto1::kan ade6-M216 his3-D1 leu1-32 ura4-D18	
15445	h? Rif1::nat mto1::kan ade6-M his3-D1 leu1-32 ura4-D18	
15454	h? rif1:nat s-rif1 mto1::kan ade6-M his3-D1 leu1-32 ura4-D18	
15469	H? taz1::hyg mto1::kan ade6-M his3-D1 leu1-32 ura4-D18	
15473	h? taz1::hyg rif1::nat mto1::kan ade6-M his3-D1 leu1-32 ura4-D18	
15471	h? taz1::hyg rif1:nat s-rif1 mto1::kan ade6-M his3-D1 leu1-32 ura4-D18	
23331	h? nup60::kan ade6-M his3-D1 leu1-32 ura4-D18	
23482	h? taz1::hyg nup60::kan ade6-M his3-D1 leu1-32 ura4-D18	
24510	h? nup132::kan ade6-M? his3-D1 leu1-32 ura4-D18	
24505	h? taz1::hyg nup132::kan ade6-M? his3-D1 leu1-32 ura4-D18	
24593	h? rif1::nat nup60::kan ade6-M? his3-D1 leu1-32 ura4-D18	
24587	h? taz1::hyg rif1::nat nup60::kan ade6-M? his3-D1 leu1-32 ura4-D18	
23447	h- nup60::kan mto1::nat ade6-M? his3-D1 leu1-32 ura4-D18	
23483	h? taz1::hyg nup60::kan mto1::nat ade6-M his3-D1 leu1-32 ura4-D18	
24688	h? mto1: mto1-427-GFP kanMX6 ade6-M his3-D1 leu1-32 ura4-D18	
24701	h? taz1::hyg mto1:mto1(1-1075)-GFPkanMX6 ade6-M his3-D1 leu1-32 ura4-D18	
24694	h? rif1::nat mto1:mto1-427-GFP kanMX6 ade6-M his3-D1 leu1-32 ura4-D18	
24695	h? taz1::hyg rif1::nat mto1:mto1-427-GFPkanMX6 ade6-M his3-D1 leu1-32 ura4-D18	
24700	h? mto1:mto1(1-1065)-GFP kanMX6 ade6-M his3-D1 leu1-32 ura4-D18	

19949	h? taz1::hyg mto1:mto1(1-1065)-GFPkanMX6 ade6-M his3-D1 leu1-32 ura4-D18	
19952	h? rif1::nat mto1:mto1(1-1065)-GFPkanMX6 ade6-M his3-D1 leu1-32 ura4-D18	
20014	h? taz1::hyg mto1:mto1(1-1065)-GFP kanMX6 rif1::nat ade6-M his3-D1 leu1-32 ura4-D18	
9317	h? rif1:kan-nmt41-GFP-Rif1 ade6-M? his3-D1 leu1-32 ura4-D18	Lab stock
8295	h- int pREP4X:SV40-NLS-GFP-lacZ:ura4+ ade6-M216 leu1-32 ura4-D18	Sazer lab
15303	rad11:rad11-GFP-kan aur1:aur1R pnda3-mcherry-atb2 ura4-D18 ade6-M leu1-32 his3-D1	
24775	taz1::nat rad:rad11-GFP-kan aur1:aur1R pnda3-mcherry-atb2 ade6-M? his3-D1 leu1-32 ura4-D18	
24768	nup60::hyg taz1::nat rad:rad11-GFP-kan aur1:aur1R pnda3-mcherry-atb2 ade6-M? his3-D1 leu1-32 ura4-D18	
24763	rif1::hyg taz1::nat rad:rad11-GFP-kan aur1:aur1R pnda3-mcherry-atb2 ade6-M? his3-D1 leu1-32 ura4-D18	
26728	taz1::hyg rqh1:rqh1-SM rad11:rad11-GFP-kan aur1:aur1R pnda3-mcherry-atb2 ura4-D18 ade6-M leu1-32 his3-D1 (cln27)	
22851	hht1:hht1-RFP-kan aur:aur1R ade6-216 ura4-D18 leu1-32 his3?	
22867	taz1::hyg hht1:hht1-RFP-kan aur:aur1R ade6-216 ura4-D18 leu1-32 his3?	
24423	nup60::kan hht1:hht1-RFP-kan aur:aur1R ade6-216 ura4-D18 leu1-32 his3?	
24428	taz1::hyg nup60::kan hht1:hht1-RFP-kan aur:aur1R ade6-216 ura4-D18 leu1-32 his3?	
22869	Mto1::kan hht1:hht1-RFP-kan aur:aur1R ade6-216 ura4-D18 leu1-32 his3?	
22854	taz1::hyg rif1::nat hht1:hht1-RFP-kan aur:aur1R ade6-216 ura4-D18 leu1-32 his3?	
22872	taz1::hyg rif1::nat mto1::kan hht1:hht1-RFP-kan aur:aur1R ade6-216 ura4-D18 leu1-32 his3?	
23336	nup60:nup60-mcherry-kanMx6 aur1:aur1R pnda1-GFP-atb2 ade6-M? his3-D1 leu1-32 ura4-D18	
23339	taz1::hyg nup60:nup60-mcherry-kanMx6 aur1:aur1R pnda1-GFP-atb2 ade6-M? his3-D1 leu1-32 ura4-D18	
23343	rif1::nat taz1::hyg nup60:nup60-mcherry-kanMx6 aur1:aur1R pnda1-GFP-atb2 ade6-M? his3-D1 leu1-32 ura4-D18	
23369	mto1::kan nup60:nup60-mcherry kan aur1:aur1R pnda1-GFP-atb2 ade6-M? his3? leu1-32 ura4-D18	
24709	mto1-427-GFP:kanMX6 nup60:nup60-mcherry-kan aur1:aur1R-pnda3-GFP-atb2 ade6-M his3-D1 leu1-32 ura4-D18	

24706	mto1(1-1065)-GFP:kanMX6 nup60:nup60-mcherry-kan aur1:aur1R-pnda3-GFP-atb2 ade6-M his3-D1 leu1-32 ura4-D18	
24771	mto1::kan rif1::hyg nup60: nup60-mcherry kan aur1:aur1R pnda1-GFP-atb2 ade6-M? his3? leu1-32 ura4-D18	
23301	int pREP4X:SV40NLS-GFP-beta-Gal-ura4+aur1:aur1R pnda3-mcherry-atb2 ura4-D18 ade6-M? leu1-132	
23304	taz1::hyg pREP4X:SV40NLS-GFP-beta-Gal-ura4+aur1:aur1R pnda3-mcherry-atb2 ura4-D18 ade6-M? leu1-32	
23311	taz1::hyg rif1::nat pREP4X:SV40NLS-GFP-beta-Gal-ura4+aur1:aur1R pnda3-mcherry-atb2 ura4-D18 ade6-M leu1-32	
20012	taz1::nat nup60::kan pREP4X:SV40NLS-GFP-beta-Gal-ura4+aur1:aur1R pnda3-mcherry-atb2 ura4-D18 ade6-M leu1-32	
13503	mto1::nat pREP4X:SV40NLS-GFP-beta-Gal-ura4+aur1:aur1R pnda3-mcherry-atb2 ura4-D18 ade6-M leu1-32	
24477	mto1::nat nup60::kan pREP4X:SV40NLS-GFP-beta-Gal-ura4+aur1:aur1R pnda3-mcherry-atb2 ura4-D18 ade6-M leu1-32	
24502	rif1::hyg mto1::nat pREP4X:SV40NLS-GFP-beta-Gal-ura4+aur1:aur1R pnda3-mcherry-atb2 ura4-D18 ade6-M leu1-32	
20026	taz1::kan mto1::nat pREP4X:SV40NLS-GFP-beta-Gal-ura4+aur1:aur1R pnda3-mcherry-atb2 ura4-D18 ade6-M leu1-32	
26716	taz1::hyg mto1::nat nup60::kan pREP4X:SV40NLS-GFP-beta-Gal-ura4+aur1:aur1R pnda3-mcherry-atb2 ura4-D18 ade6-M leu1-32	
	Mto1D spindle persistence	
	Mto1D nup60D spindle persistence	
24444	man1:man1-tdTomato-hphR rif1:kan nmt41-GFP-rif1 ade6-M His? leu1-32 ura4-D18	
24441	nup60:nup60-mcherry-kan rif1:kan nmt41-GFP-rif1 ade6-M His? leu1-32 ura4-D18	
26722	nup60::nat rif1:kan-nmt41-GFP-rif1 dis2::kan ade6-M? his3-D1 leu1-32 ura4-D18	
23388	h- nup60:nup60-13myc Nat ade6-M210 his3-D1 leu1-32 ura4-D18	
23430	h- nup60:nup60-13myc Nat taz1::kan ade6-M210 his3-D1 leu1-32 ura4-D18	
23427	h- nup60:nup60-13myc Nat rif1::hyg ade6-M210 his3-D1 leu1-32 ura4-D18	
23433	h- nup60:nup60-13myc Nat taz1::kan rif1::hyg ade6-M210 his3-D1 leu1-32 ura4-D18	

15316	h- cut11:cut11-13myc-nat ura4-D18 ade6-M leu1-32 his3-D1	
24599	h? taz1::hyg cut11:cut11-13myc-nat ura4-D18 ade6-M leu1-32 his3-D1	
1337	h? rap1:rap1-13myc-kanMX	
15306	h? rap1:rap1-13myc-kanMX taz1::nat	
26736	h? nup60:nup60-13myc-nat ade6-M? leu1-32 his3-D1	
26740	h? nup60:nup60-13myc-nat top2:top2-191 ade6-M? his3-D1	
1226	h+ taz1::hyg rad11:rad11-mCherry-kan lys1+:pbqt4-GFP-bqt4 leu1-32 ura4-D18 ade6	Lab stock
24651	h? rif1:rif1-myc-GFP-kan nup60:nup60-mcherry-kan ade6-M his3-D1 leu1-32 ura4-D18	
24677	h? taz1::hyg rif1:rif1-myc-GFP-kan ade6-M his3-D1 leu1-32 ura4-D18	
24472	nup60::kan pREP4X:SV40NLS-GFP-beta-Gal-ura4+aur1:aur1R pnda3-mcherry-atb2 ura4-D18 ade6-M leu1-32	
18435	h+ imp1::kan ade6-M216 his3-D1 leu1-32 ura4-D18	
23335	h- taz1::hyg ade6-M? his3-D1 leu1-32 ura4-D18	
26672	h? nup60:nup60-mcherry-kan lys1+-GFP-bqt4 leu1-32 ura4-D18 ade6-M? his3-D1	
26676	h? aur1:aur1R pnda3-mcherry-atb2 lys1+-GFP-bqt4 leu1-32 ura4-D18 ade6-M?	
20020	h? imp1::kan nup60:nup60-mcherry-kan aur1:aur1R-pnda3-GFP-atb2 ade6-M his3-D1 leu1-32 ura4-D18	
15496	h- aur1R-Pnda3-GFP-atb2	Lab stock
