# Supplementary material for: Fate of telomere entanglements is dictated by the timing of anaphase midregion nuclear envelope breakdown
Source: Nat Commun. 2024 Jun 3;15:4707. doi: 10.1038/s41467-024-48382-2 (PMC11148042; doi:10.1038/s41467-024-48382-2)
Supplement: Supplementary file 3 — Description of Additional Supplementary Files [file 41467_2024_48382_MOESM3_ESM.pdf]

Description of Additional Supplementary Files

File Name: Supplementary Data 1
Description: List of oligonucleotides used in the current study.

File Name: Movie S1
Description: Time lapse video of a mitotically dividing cell lacking Taz1 maintained at 32°C, expressing GFP-Bqt4 (NE marker) and Rad11-mcherry (RPA), example #1.

File Name: Movie S2
Description: Time lapse video of a mitotically dividing cell lacking Taz1 maintained at 32°C, expressing GFP-Bqt4 (NE marker) and Rad11-mcherry (RPA), example #2.

File Name: Movie S3
Description: Time lapse video of a mitotically dividing cell lacking Taz1 maintained at 32°C, expressing GFP-Bqt4 (NE marker) and Rad11-mcherry (RPA), example #3.

File Name: Movie S4
Description: Time lapse video of a mitotically dividing cell lacking Taz1 maintained at 32°C, expressing GFP-Bqt4 (NE marker) and Rad11-mcherry (RPA), example #4.
