# Supplementary material for: Fate of telomere entanglements is dictated by the timing of anaphase midregion nuclear envelope breakdown
Source: Nat Commun. 2024 Jun 3;15:4707. doi: 10.1038/s41467-024-48382-2 (PMC11148042; doi:10.1038/s41467-024-48382-2)
Supplement: Supplementary file 9 — Reporting Summary [file 41467_2024_48382_MOESM9_ESM.pdf]

nature portfolio

Corresponding author(s): Julia Promisel Cooper, Rishi Kumar Nageshan

Last updated by author(s): Feb 27, 2024

Reporting Summary

Nature Portfolio wishes to improve the reproducibility of the work that we publish. This form provides structure for consistency and transparency in reporting. For further information on Nature Portfolio policies, see our Editorial Policies and the Editorial Policy Checklist.

Statistics

For all statistical analyses, confirm that the following items are present in the figure legend, table legend, main text, or Methods section.

n/a	Confirmed	
☐	☒	The exact sample size (*n*) for each experimental group/condition, given as a discrete number and unit of measurement
☐	☒	A statement on whether measurements were taken from distinct samples or whether the same sample was measured repeatedly
☐	☒	The statistical test(s) used AND whether they are one- or two-sided<br>*Only common tests should be described solely by name; describe more complex techniques in the Methods section.*
☒	☐	A description of all covariates tested
☐	☒	A description of any assumptions or corrections, such as tests of normality and adjustment for multiple comparisons
☐	☒	A full description of the statistical parameters including central tendency (e.g. means) or other basic estimates (e.g. regression coefficient) AND variation (e.g. standard deviation) or associated estimates of uncertainty (e.g. confidence intervals)
☐	☒	For null hypothesis testing, the test statistic (e.g. *F*, *t*, *r*) with confidence intervals, effect sizes, degrees of freedom and *P* value noted<br>*Give P values as exact values whenever suitable.*
☒	☐	For Bayesian analysis, information on the choice of priors and Markov chain Monte Carlo settings
☒	☐	For hierarchical and complex designs, identification of the appropriate level for tests and full reporting of outcomes
☒	☐	Estimates of effect sizes (e.g. Cohen's *d*, Pearson's *r*), indicating how they were calculated

Our web collection on statistics for biologists contains articles on many of the points above.

Software and code

Policy information about availability of computer code

Data collection	Image collection was performed with SoftWorx (GE Healthcare Life Sciences, version 7.2.1 and version 7.2.2)
Data analysis	Statistical analyses were performed with Prism (GraphPad Software Inc., version 10.1.0); images were analyzed with Fiji/ImageJ (open source, versions 2.1.0/1.53c); Spindle, midregion stability, NPC disassembly and viability data were compiled in Microsoft Excel (Microsoft, version 16.82).

For manuscripts utilizing custom algorithms or software that are central to the research but not yet described in published literature, software must be made available to editors and reviewers. We strongly encourage code deposition in a community repository (e.g. GitHub). See the Nature Portfolio guidelines for submitting code & software for further information.

Data

Policy information about availability of data

All manuscripts must include a data availability statement. This statement should provide the following information, where applicable:

- Accession codes, unique identifiers, or web links for publicly available datasets
- A description of any restrictions on data availability
- For clinical datasets or third party data, please ensure that the statement adheres to our policy

Raw data are available in the Source Files; there are no restrictions on data availability.

Research involving human participants, their data, or biological material

Policy information about studies with human participants or human data. See also policy information about sex, gender (identity/presentation), and sexual orientation and race, ethnicity and racism.

Reporting on sex and gender	N/A
Reporting on race, ethnicity, or other socially relevant groupings	N/A
Population characteristics	N/A
Recruitment	N/A
Ethics oversight	N/A

Note that full information on the approval of the study protocol must also be provided in the manuscript.

Field-specific reporting

Please select the one below that is the best fit for your research. If you are not sure, read the appropriate sections before making your selection.

[x] Life sciences [ ] Behavioural & social sciences [ ] Ecological, evolutionary & environmental sciences

For a reference copy of the document with all sections, see nature.com/documents/nr-reporting-summary-flat.pdf

Life sciences study design

All studies must disclose on these points even when the disclosure is negative.

Sample size	Sample size was as large as practicable for all experiments.
Data exclusions	No data was excluded.
Replication	All the microscopy based data were collected from two to three independent experiments with two different clones. A corresponding wildtype strain that was run in parallel with each experiment served as a control. Growth experiments are repeated three to five times with at least two different clones.
Randomization	Randomization is built in to all of our experiments, as colonies are chosen randomly for outgrowth.
Blinding	Blinding was unnecessary as the phenotypes were obvious, with only exception for the data represented in the Figure S4D which was collected by double blind experiments.

Reporting for specific materials, systems and methods

We require information from authors about some types of materials, experimental systems and methods used in many studies. Here, indicate whether each material, system or method listed is relevant to your study. If you are not sure if a list item applies to your research, read the appropriate section before selecting a response.

Materials & experimental systems

n/a	Involved in the study	
[ ]	[x]	Antibodies
[x]	[ ]	Eukaryotic cell lines
[x]	[ ]	Palaeontology and archaeology
[x]	[ ]	Animals and other organisms
[x]	[ ]	Clinical data
[x]	[ ]	Dual use research of concern
[x]	[ ]	Plants

Methods

n/a	Involved in the study	
[x]	[ ]	ChIP-seq
[x]	[ ]	Flow cytometry
[x]	[ ]	MRI-based neuroimaging

Antibodies

Antibodies used	GFP antibody (ChromoTek, Cat no: H39, Lot no:104272-02-02); Anti-Rat-HRP conjugate Goat antibody (Vector Labs, Cat no: PI-9400, Lot no: ZH0820)

Validation	The anti-GFP antibody was validated with a negative and positive controls.

Plants

Seed stocks	N/A
Novel plant genotypes	N/A
Authentication	N/A
